# Trench Foot or Non-Freezing Cold Injury As a Painful Vaso-Neuropathy: Clinical and Skin Biopsy Assessments

**DOI:** 10.3389/fneur.2017.00514

**Published:** 2017-09-29

**Authors:** Praveen Anand, Rosario Privitera, Yiangos Yiangou, Philippe Donatien, Rolfe Birch, Peter Misra

**Affiliations:** ^1^Peripheral Neuropathy Unit, Centre for Clinical Translation, Hammersmith Hospital, Imperial College, London, United Kingdom

**Keywords:** trench foot, non-freezing cold injury, neuropathy, pain, skin biopsy, nerve conduction study

## Abstract

**Background:**

Trench foot, or non-freezing cold injury (NFCI), results from cold exposure of sufficient severity and duration above freezing point, with consequent sensory and vascular abnormalities which may persist for years. Based on observations of Trench foot in World War II, the condition was described as a vaso-neuropathy. While some reports have documented nerve damage after extreme cold exposure, sensory nerve fibres and vasculature have not been assessed with recent techniques in NFCI.

**Objective:**

To assess patients with chronic sensory symptoms following cold exposure, in order to diagnose any underlying small fibre neuropathy, and provide insight into mechanisms of the persistent pain and cold hypersensitivity.

**Methods:**

Thirty soldiers with cold exposure and persistent sensory symptoms (>4 months) were assessed with quantitative sensory testing, nerve conduction studies, and skin biopsies. Immunohistochemistry was used to assess intraepidermal (IENF) and subepidermal (SENF) nerve fibres with a range of markers, including the pan-neuronal marker protein gene product 9.5 (PGP 9.5), regenerating fibres with growth-associated protein 43 (GAP43), and nociceptor fibres with transient receptor potential cation channel subfamily V member 1 (TRPV1), sensory neuron-specific receptor (SNSR), and calcitonin gene-related peptide (CGRP). von Willebrand factor (vWF), endothelial nitric oxide synthase (eNOS), and vascular endothelial growth factor (VEGF) were used for assessing blood vessels, and transient receptor potential cation channel, subfamily A member 1 (TRPA1) and P2X purinoceptor 7 (P2X7) for keratinocytes, which regulate nociceptors *via* release of nerve growth factor.

**Results:**

Clinical examination showed pinprick sensation was abnormal in the feet of 20 patients (67%), and between 67 and 83% had abnormalities of thermal thresholds to the different modalities. 7 patients (23%) showed reduced sensory action potential amplitude of plantar nerves. 27 patients (90%) had decreased calf skin PGP 9.5 IENF (*p* < 0.0001), the remaining 3 patients had decreased nerve markers in subepidermis or foot skin. There were marked increases of all vascular markers (for vWF in calf skin, *p* < 0.0001), and increased sensory or regenerating SENF (for calf skin, GAP43, *p* = 0.002). TRPA1 (*p* = 0.0012) and P2X7 (*p* < 0.0001) were increased in basal keratinocytes.

**Conclusion:**

A range of skin biopsy markers and plantar nerve conduction studies are useful objective assessments for the diagnosis of peripheral neuropathy in NFCI. Our results suggest that an increase in blood vessels following tissue ischaemia/hypoxia could be associated with disproportionate and abnormal nerve fibres (irritable nociceptors), and may lead to NFCI as a “painful vaso-neuropathy.”

## Introduction

Trench foot or non-freezing cold injury (NFCI) has been documented historically in a number of military conflicts (1). Clinical observations and possible pathophysiological mechanisms were described in World Wars I and II, when Trench foot became a major problem (2). During this period, key studies were conducted by Hughes (3) in soldiers operating in trenches, and by Ungley et al. (4–7), in soldiers who had prolonged limb immersion in cold sea water. However, despite efforts for its prevention, NFCI remains a significant current clinical problem in military personnel, and in some civilians (fishermen, cold storage workers, skiiers, and mountaineers).

Ungley and Blackwood (4) described the non-freezing or cold immersion syndrome as dominated by neurovascular changes, and designated the condition a “peripheral vasoneuropathy after chilling.” Based on these observations, the sequence of events was classified into three stages. In the first stage (“pre-hyperaemic,” stage 1), lasting few hours to several days, the extremities are cold, numb in a glove and sock distribution, swollen, and discoloured. In severe cases, arterial pulses are absent and remain so in severe cases going on to gangrene. In the following stage (“hyperaemic,” stage 2), which may last 6–10 weeks, the clinical features are vascular (red hot skin), sensory (tingling, stabbing pain, cold hypersensitivity), along with increased swelling. In the late chronic stage (“post-hyperaemic,” stage 3), symptoms such as pain, numbness, and cold hypersensitivity are prominent, sometimes persisting for many years, along with sweating and trophic abnormalities in some cases.

During World War II, the severity of NFCI was graded clinically for the above stages (8). The grades were as follows: minimal injury (grade A), with hyperaemia and slight sensory changes, with occasional cold hypersensitivity; mild injury (grade B), characterised by oedema, hyperaemia, definite sensory changes, neuropathic pain, cold hypersensitivity, but no skin bullae; moderate injury (grade C), dominated by oedema, hyperaemia, bullae and mottling, pronounced anaesthesia and cold hypersensitivity; severe injury (grade D), defined by the presence of severe oedema, extravasation of blood, and incipient gangrene.

While preventive measures have reduced its incidence and severity, NFCI is still the most frequent non-combat related military injury; many cases were reported during the Falkland conflict (9), and in Afghanistan (10). While World War II tissue studies documented nerve damage (11–14), including of plantar nerves, these were in severe cases. Cutaneous sensory nerve fibres and vasculature have not been studied with recent histological techniques, which would be particularly useful in mild NFCI stage 3, the most common current clinical presentation.

We have assessed 30 soldiers referred to our Peripheral Neuropathy Clinic, with a history of cold exposure and persistent symptoms, in accord with NFCI (stage 3, grades A–B). The assessments included quantitative sensory testing (QST), nerve conduction studies, and skin punch biopsies. The aim was to diagnose any underlying painful vaso-neuropathy, particularly with objective techniques. We and others have previously shown that a range of diagnostic markers are useful in skin biopsies, including protein gene product 9.5 (PGP 9.5) (pan-neuronal), transient receptor potential cation channel subfamily V member 1 (TRPV1) (heat and capsaicin receptor), calcitonin gene-related peptide (CGRP) and sensory neuron-specific receptor (SNSR) (nociceptor subsets), growth-associated protein 43 (GAP43) (regenerating nerve fibres), and vascular markers (e.g., von Willebrand factor, vWF) (15–18). We have previously reported the localisation and function of transient receptor potential cation channel, subfamily A member 1 (TRPA1) (19) and P2X purinoceptor 7 (P2X7) receptors (20) in tissues from patients with neuropathic pain conditions. While intraepidermal nerve fibre (IENF) density with PGP 9.5 is standard, we routinely assess other nerve and vascular markers, which may be more sensitive in some conditions [e.g., advanced painful diabetic neuropathy (18)], and indicate the underlying cause, e.g., neuro-inflammation. Importantly, accurate diagnosis of small fibre neuropathy provides guidance for specific symptomatic and disease-modifying treatments, and markers to investigate the time-course and clinical outcomes.

## Materials and Methods

### Participants

Thirty soldiers were referred by UK military and civilian General Practitioners, and a specialist War Injury Clinic, with a provisional diagnosis of NFCI. They were assessed at the Peripheral Neuropathy Clinic, Hammersmith Hospital, Imperial College Healthcare NHS Trust, according to standard NHS Trust clinical practice. The assessments included clinical examination, QST, nerve conduction studies, and skin punch biopsies. All soldiers gave their written informed consent for skin biopsy. The data presented here are the outcome of an internal Departmental audit, which reviewed the assessment of patients.

The mean age of subjects was 31 years (range 20–44 years); 20 were African/Caribbean males, 9 were Caucasian males, and 1 was a Caucasian female. All had a past medical history of cold exposure during military activities with persistent sensory symptoms, despite wearing protective gear and following preventive measures. Fifteen soldiers (50%) reported symptoms in hands and feet, 1 soldier (3%) only in hands, and 14 soldiers (47%) only in feet. Twenty-six subjects (86%) reported pain symptoms worsened by cold exposure including burning pain, one subject reported pulsating pain, and one subject stabbing pain. The remaining subjects reported other sensory symptoms. Pins and needles were reported by 19 subjects (63%), itching by 3 subjects (13%), and electric shock sensations by 3 subjects (10%). All subjects described their extremities as cold and difficult to rewarm. Six subjects (20%) reported changes in colour of the feet, one (3%) in the hands, while five subjects (16%) reported swelling of the extremities.

All subjects were initially diagnosed with a mild NFCI (grade B). One soldier was diagnosed with a minimal NFCI (grade A) in the hands and mild NFCI (grade B) in the feet.

The assessment at our Peripheral Neuropathy Clinic conducted was conducted after a mean duration of 27 months (range 4 months to 9 years). The subjects reported their symptoms as persistent since onset and significant. They were at the post-hyperaemic stage 3, grade B. Seventeen subjects (56%) were taking medications for pain symptoms, including amitriptyline (*n* = 10), gabapentin (*n* = 1), pregabalin (*n* = 1), duloxetine (*n* = 1), ibuprofen (*n* = 2), paracetamol (*n* = 1), and lidocaine 5% patch (*n* = 1).

### Quantitative Sensory Testing

Thresholds for light touch were measured using Semmes–Weinstein hairs (made by A. Ainsworth, University College London, UK), No. 1 (0.0174 g) to No. 20 (263.0 g). The number of the hair with the lowest force reliably detected by the patient on the dorsum of the great toe was recorded. Values >No. 3 monofilament (0.0479 g) were considered abnormal (15). Dynamic allodynia was tested by gently stroking the skin with a standardised yellow brush (Somedic, Stockholm, Sweden).

Vibration perception thresholds were measured using a biothesiometer (Biomedical Instrument Company, Newbury, OH, USA), placed on the distal index finger and great toe joints. Three ascending and three descending trials were carried out, and the mean value obtained. Values >12 V were considered abnormal (21, 22).

Thermal perception thresholds were performed as described previously (21, 22), using the TSA II–NeuroSensory Analyser (Medoc, Ramat Yishai, Israel). A 30 mm × 30 mm thermode was used and thermal thresholds determined in the soles of the feet (instep) for warmth perception, cool perception, heat pain, and cold pain, from a baseline temperature of 32°C, with a change in temperature of 1°C/s. The mean of three consecutive tests for each modality was recorded. Values >6.4°C for warm sensation, >2.3°C for cool sensation, and >10.4°C for heat pain were considered abnormal (21, 22).

### Neurophysiological Assessments

Nerve conduction and sympathetic skin response (SSR) studies were performed by the same examiner on a Medtronic Keypoint electromyogram system (Medtronic, Minneapolis, MN, USA) using standard techniques. Antidromic sensory action potentials for the sural and superficial peroneal nerves and orthodromic sensory action potentials for the medial and lateral plantar, median, and ulnar nerves on both sides were recorded.

For the sural, superficial peroneal, medial plantar, and ulnar nerves, action potentials of <5 μV amplitude were considered abnormal, while for the lateral plantar and median nerves, amplitudes of <2.5 μV and <10 μV were considered abnormal, respectively. Sensory conduction velocities of <50 and 40 m/s were considered abnormal for upper and lower limb nerves, respectively. SSRs were recorded from both palms and both soles in response to a sudden unexpected tactile stimulus and a sudden inspiratory gasp. An absent SSR was considered abnormal.

### Calf Skin Biopsy and Immunohistochemistry

Two 3.5-mm-diameter skin punch biopsies were collected under local anaesthetic from the lateral calf, and one biopsy from the dorsum of the foot, between the great and second toes. Only one biopsy was done from the foot to enable subjects to travel from the clinic in their usual footwear with ease. One subject did not have a biopsy from the dorsum of the foot for logistical reasons. Skin biopsies were processed as described previously (17). One of the two calf skin biopsies was snap frozen [for the sensory neuronal marker TRPV1, vascular markers vWF, endothelial nitric oxide synthase (eNOS), and vascular endothelial growth factor (VEGF)] and stored at −70°C. The other calf biopsy and the foot biopsy were immersed in fixative [modified Zamboni’s fluid—2% formalin; 0.01 M phosphate buffer; 15% saturated picric acid (pH 7.2)] and then washed in phosphate-buffered saline (PBS; 0.1 M phosphate; 0.9% w/v saline; pH 7.3) containing 15% w/v sucrose for an hour, before snap freezing in optimum cutting tissue embedding medium (Tissue-Tek OCT, RA Lamb Ltd., Eastbourne, UK). These were used for pan-neuronal marker PGP 9.5, sensory nerve sub-set markers SNSR and CGRP, regenerating nerve fibre marker GAP43, cold nociceptor TRPA1 (19), endothelial vascular marker cluster of differentiation 31 (CD31), and P2X7 receptor (20).

Unfixed, frozen sections (15 µm thickness for all except GAP43 at 30 µm) were collected onto poly-l-lysine- (Sigma, Poole, UK) coated glass slides and post-fixed in freshly prepared, 4% w/v paraformaldehyde in 0.15 M PBS for 30 min. Frozen sections of pre-fixed (Zamboni’s) were collected in the same way and allowed to air dry. Endogenous peroxidase was blocked by incubation in methanol containing 0.3% w/v hydrogen peroxide for 30 min for both post- and pre- (Zamboni) fixed sections. After rehydration, sections were incubated overnight with primary antibody (Table 1). Sites of primary antibody attachment were revealed using nickel-enhanced, avidin–biotin peroxidase (ABC—Vector Laboratories, Peterborough, UK) as previously described (23, 24). Sections were counterstained for nuclei in 0.1% w/v aqueous neutral red, air dried, and mounted in xylene-based mountant (DPX; BDH/Merck, Poole, UK), prior to analysis.

**Table 1:** List of antibodies used in this study.

Antibodies to	Source	Ref #	Titre
Protein gene product 9.5	Ultraclone, Isle of Wight, UK	RA101	1:40,000
Transient receptor potential cation channel subfamily V member 1	GlaxoSmithKline, Harlow, UK	C22	1:10,000
Sensory neuron-specific receptor	Cambridge Research Biochemicals, Billingham, UK	MRV2	1:15,000
Growth-associated protein 43	Sigma, Poole, UK	Clone 7B10	1:80,000
von Willebrand factor	Novocastra Laboratories, Milton Keynes, UK	NCL-vWFp	1:10,000
Vascular endothelial growth factor	Santa Cruz Biotechnology, Inc., Dallas, TX, USA	SC-152	1:2,500
Endothelial nitric oxide synthase	Santa Cruz Biotechnology, Inc., Dallas, TX, USA	SC-654	1:500
Cluster of differentiation 31	Dakocytomation, Dako UK, Ltd., Cambridge, UK	Clone JC70A	1:200
		M0823	
Calcitonin gene-related peptide	Chemicon/Millipore, Feltham, UK	AB5920	1:4,000
Transient receptor potential cation channel, subfamily A member 1	GlaxoSmithKline, Harlow, UK	1962	1:1,000
P2X purinoceptor 7 receptor	Geneva Biomedical Research Institute, Geneva, Switzerland	EL101116/122	1:64,000

50-µm sections were also studied with PGP 9.5 antibody. Briefly, fixed sections were floated onto PBS in 12-well plates, dehydrated with alcohol/hydrogen peroxide solution for 30 min, washed with PBS and incubated with PGP 9.5 overnight, washed and incubated with second antibody for 1 h, and then washed and incubated with ABC as above. After washing, nickel developer solution was added and staining allowed to develop. The reaction was stopped by adding 0.1 M sodium acetate pH 6.0, washed again in PBS, counterstained and free floated onto PPL slides, allowed to dry and incubated in xylene, and finally mounted using DPX mountant.

The immunohistological methods and specificity of all antibodies used other than SNSR have been reported previously (17, 23–25). The SNSR antibody was a gift from Dr D. O’Donnell (AstraZeneca, Montreal, Canada), and its characterisation is described below. Negative controls included omission of primary antibodies or their replacement with preimmune serum.

A custom polyclonal antibody to human SNSR4 denoted “MRV2,” was generated by Cambridge Research Biochemicals (Cleveland, United Kingdom). A candidate peptide corresponding to amino acids 307–322 (located in the intracellular C-terminal region) was chosen because it was unique to human SNSR4. Three rabbits were immunised with a 16-mer polypeptide antigen coupled to keyhole limpet haemocyanin *via* cysteine residues. The resulting serum was affinity purified using glycine elution. Antibody specificity was confirmed by immunocytochemistry in Hek 293 cells cotransfected with hSNSR4 and the synthetic fusion protein, FLAG (DYKDDDDK). Selectivity of the MRV2 antibody was further confirmed using two additional SNSR subtypes. Hek293 cells were transfected with either SNSR5 or SNSR6 and assessed by immunocytochemistry as outlined above. The specificity and co-labelling with IB-4 were also confirmed in human DRG and spinal cord. Preimmune serum was diluted to 1:5,000 before being applied to control tissue sections. Peptide preabsorption was successfully performed using 10 µg/µl homologous peptide antigen incubated with MRV2 overnight at 4°C.

For immunohistochemical colocalisation studies, serial sections of human skin were double-labelled by overnight incubation with a mixture of primary polyclonal antibody to PGP 9.5 (dilution 1:40,000) or SNSR (dilution 1:15,000) and a monoclonal antibody to the vascular endothelial cell marker CD31 (Dako Cytomation, dilution 1:200). Immunoreaction for the nerve markers was first revealed using the standard nickel-enhanced ABC peroxidase method as above to give a grey/black product. The endothelial cell marker was then incubated with biotinylated anti-mouse antibody for 1 h followed by incubation with ABC alkaline phosphatase for a further 1 h and the immunoreactivity developed with Fast Red to give a red product. Double-labelled preparations were mounted in Ultramount medium (Vector Labs UK).

As a range of nerve and vascular markers were assessed, the skin biopsy tissue volume was not sufficient for all markers in some biopsies, and precluded studies with 50-µm sections other than with the PGP 9.5 antibody—numbers different from the full set are indicated in their respective Section “Results.”

### Image Analysis

The IENF quantification method used in this study was modified from the EFNS guidelines (26) since thinner tissue sections were handled; IENF counts included all nerve fibres crossing the dermal–epidermal junction. In addition, clearly branched epidermal nerve fragments that did not cross the basement membrane were also counted. This method was previously reported by us (16, 17) and validated (27). However, 50 µm sections were also studied with PGP 9.5 antibody in 15 NFCI biopsies as sufficient tissue was available and quantified according to the EFNS guidelines. Our PGP 9.5 control values for 50 µm thickness sections are within the range of published values recommended by European Federation of Neurological Societies/Peripheral Nerve Society Guidelines [(26), and also Ref. (28–30)]. Thicker (30 µm) tissue sections were used to increase the sensitivity of GAP43 staining since these markers showed fine calibre IENF, as described previously (16, 17).

Intraepidermal fibres were counted as described above for PGP 9.5, SNSR, TRPV1, and GAP43 and expressed as fibres per millimetre length of the epidermis. For subepidermal nerve fibres (SENFs) and vascular markers, analysis to a depth of 200 µm below the basal epidermis; five sections were photographed *via* video link to an Olympus BX50 microscope, image analysis performed using analySIS FIVE software (Olympus, Watford, Hertfordshire, UK), and the mean values used for statistical analysis. TRPA1, and P2X7 expressing keratinocytes, within the entire epithelium of three sections were also measured by image analysis as above, and the mean values used for statistical analysis.

Control values have been published for the nerve markers in calf skin, where these have not been published, the numbers of matched control skin biopsies are indicated in Section “Results.” Foot skin biopsies have been analysed in comparison with calf skin biopsies in the NFCI subjects (note the density of IENF and SENF is generally lower in distal than proximal limbs).

### Statistical Analysis

Data was analysed using GraphPad Prism version 5.0 for Windows (GraphPad Prism Software, San Diego, CA, USA), Mann–Whitney *U* test, *p* < 0.05 (two-tailed) indicated significance.

## Results

### Clinical Examination

Clinical examination was unremarkable other than sensory examination in all subjects. Joint position sense at the great toe was abnormal in five subjects. Pinprick sensation was abnormal in 20 subjects (67%), and also in the hands in 17 subjects (57%).

### Quantitative Sensory Testing

Monofilament detection threshold was abnormal at the toe in 19 subjects (63%), and also in the fingers in 16 subjects (53%). No dynamic allodynia was detected during the examination with the Yellow brush.

Vibration threshold was abnormal at the toes in 12 subjects (40%), and also in the fingers in 8 subjects (27%).

Cool threshold was abnormal in the feet in 22 subjects (73%), and also in the hands in 17 subjects (57%). Warm threshold was abnormal in the feet in 25 subjects (83%), and also in the hands in 14 subjects (47%). Abnormality of cold pain thresholds were found in the feet of 20 subjects (66%), and also in the hands in 17 subjects (56%). Abnormalities of heat pain thresholds were found in the feet of 20 subjects (66%), and also in the hands in 16 subjects (53%).

### Neurophysiological Assessments

**Figure 1:**
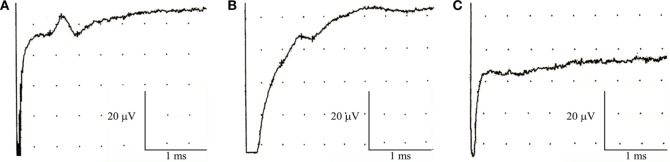
Nerve conduction studies in patients with non-freezing cold injury. Sensory nerve action potential of the plantar nerves. **(A)** Normal, **(B)** reduced amplitude, and **(C)** absent.

**Figure 2:**
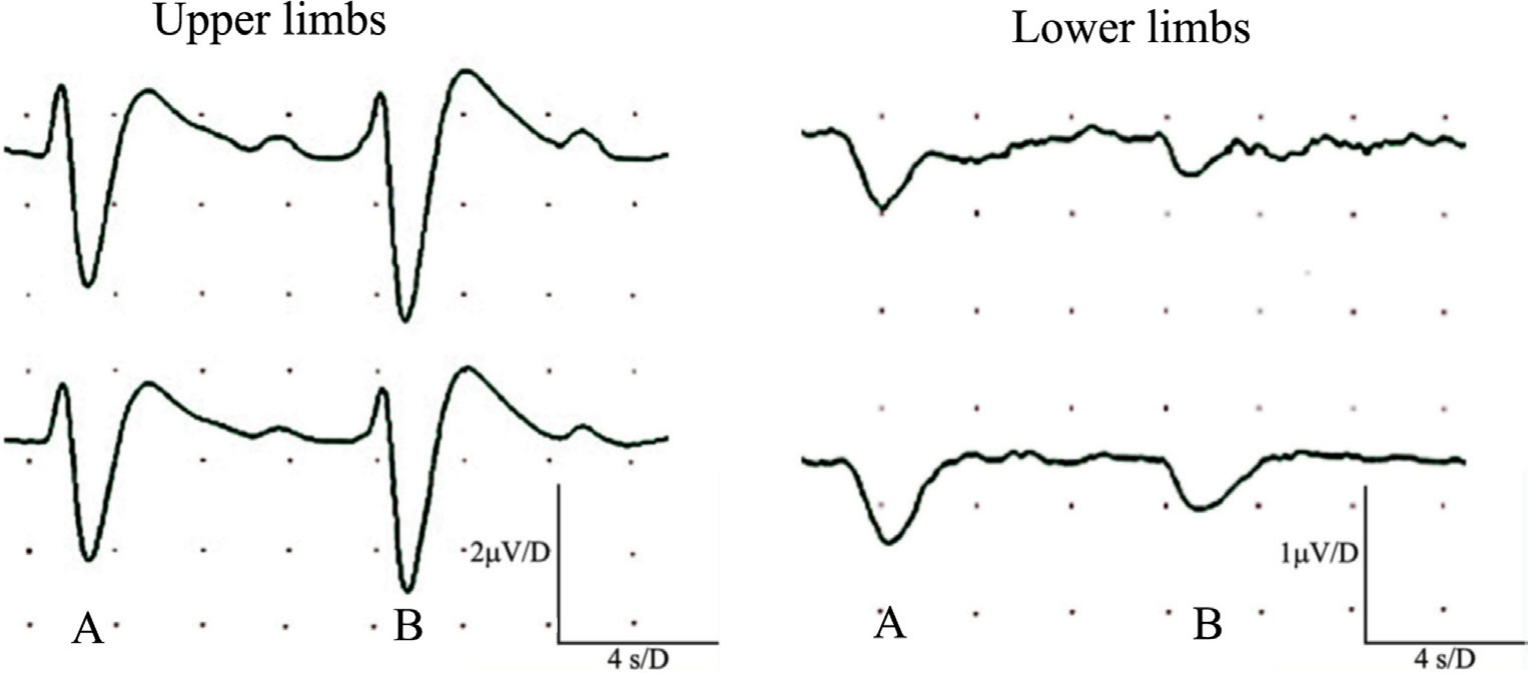
Simultaneous recordings of normal sympathetic skin responses (SSR) from both hands and feet. SSR evoked by light, sudden unexpected tactile stimulus **(A)**, and a sudden inspiratory gasp **(B)**.

Seven subjects (23%) showed abnormalities restricted to the sensory action potential amplitude of plantar nerves, at times asymmetrically (Figure 1). SSRs from the palms and soles were normal in all cases (Figure 2).

### Immunohistochemistry

#### Protein Gene Product 9.5

**Figure 3:**
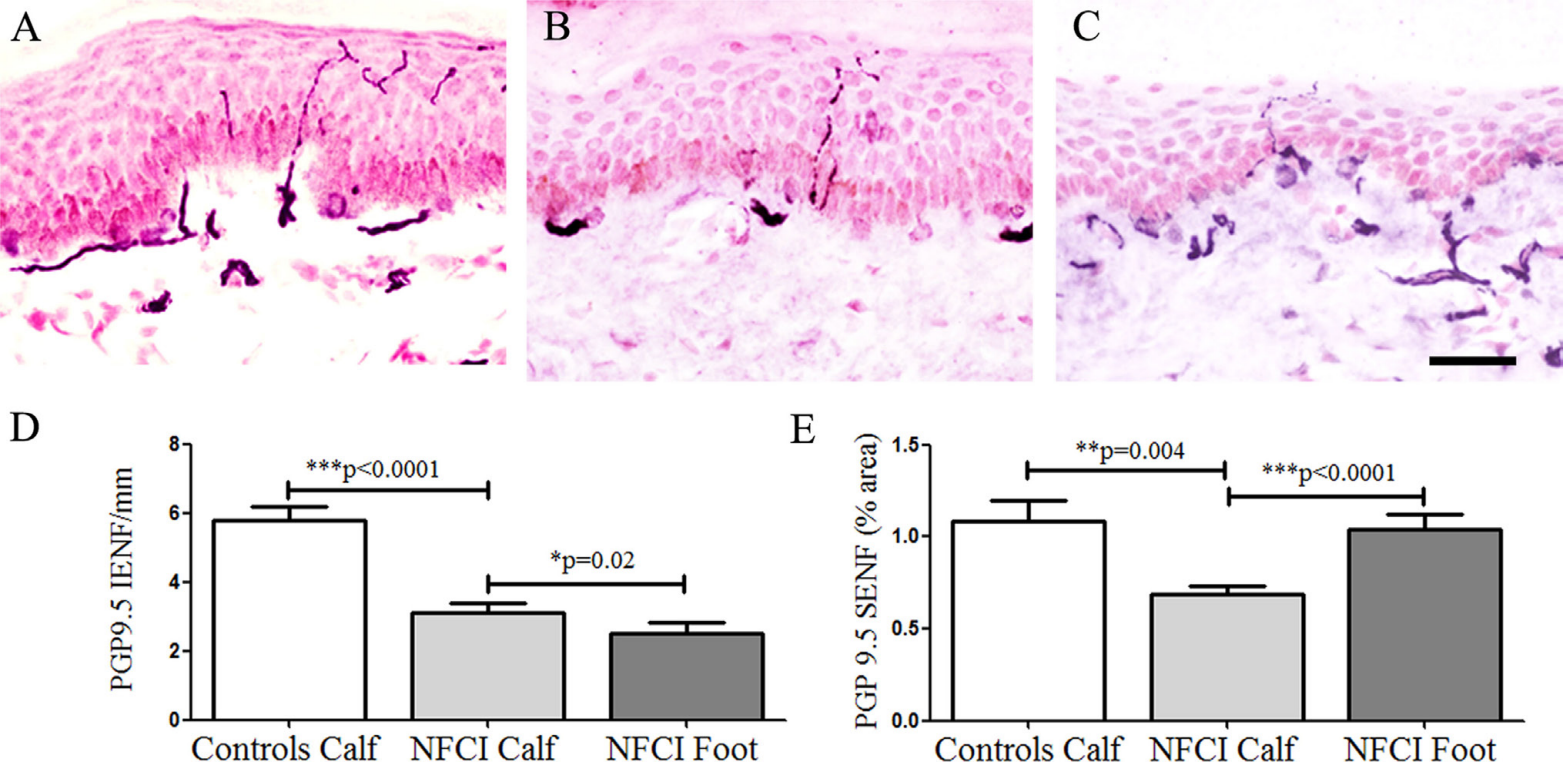
Protein gene product 9.5 (PGP 9.5) immunoreactivity in skin. PGP 9.5 staining in control calf skin **(A)**, in non-freezing cold injury (NFCI) calf skin **(B)**, and in NFCI foot skin **(C)**; scale bar = 50 µm; bar charts of the PGP 9.5 intraepidermal nerve fibre (IENF)/mm **(D)**; bar charts of PGP 9.5 subepidermal nerve fibre (SENF) (% area) **(E)**.

Protein gene product 9.5-immunoreactive IENFs in calf skin showed a significant decrease in NFCI (*p* < 0.0001; Figure 3). Image analysis of SENF PGP 9.5-immunoreactive nerve fibres in NFCI calf skin also showed a reduction (*p* = 0.004; Figure 3). 27 subjects (90%) had abnormal IENF values, and 28 subjects (93%) abnormal SENF values.

Protein gene product 9.5-immunoreactive IENF was also assessed using 50-µm calf skin section in 15 NFCI biopsies as sufficient tissue was available, and all showed low PGP 9.5 compared to controls (mean ± SEM NFCI 3.18 ± 0.54 controls 8.29 ± 0.34, *p* < 0.0001). All these 15 skin biopsies also had reduced numbers of IENF in 15 µm thickness sections.

The PGP 9.5 IENF fibres in the NFCI foot skin were significantly lower compared to NFCI calf skin (*p* = 0.02; Figure 3). There were significantly higher numbers of PGP 9.5 SENF in NFCI foot compared to NFCI calf skin (*p* < 0.0001; Figure 3).

With assessment of 15 µm skin biopsy sections, three NFCI subjects had PGP 9.5 values within our normal range; however, one of these subjects had diminished monofilament and pin prick sensation in the fingers and toes, reduced amplitude of the left lateral plantar nerve sensory action potential, decreased PGP 9.5 SENF in calf skin, and markedly decreased PGP 9.5 IENF in foot skin; another subject had diminished monofilament and pin prick sensation in the toes, decreased TRPV1 IENF in calf skin, and decreased IENF for all markers including PGP 9.5 in foot skin; the third subject had decreased diminished monofilament and pin prick sensation in the fingers and toes, and decreased SNSR IENF in foot skin.

#### Transient Receptor Potential Cation Channel Subfamily V Member 1

**Figure 4:**
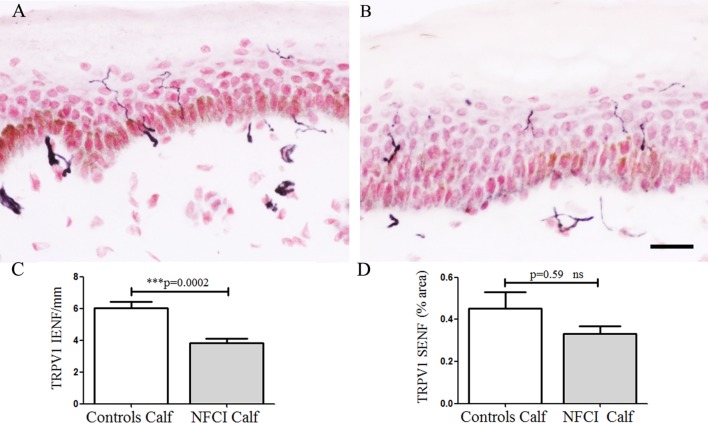
Transient receptor potential cation channel subfamily V member 1 (TRPV1) immunoreactivity in skin. TRPV1 staining in control calf skin **(A)**, in non-freezing cold injury (NFCI) calf skin **(B)**; scale bar = 50 µm; bar charts of the TRPV1 intraepidermal nerve fibre (IENF) fibres/mm **(C)**; bar charts of the image analysis of TRPV1 subepidermal nerve fibre (SENF) (% area) **(D)**.

Transient receptor potential cation channel subfamily V member 1 IENF showed a significant decrease in NFCI calf skin (*p* = 0.0002; Figure 4). IENF and SENF analysis showed 97 and 80%, respectively, of NFCI subjects, had abnormal values.

#### Sensory Neuron-Specific Receptor

**Figure 5:**
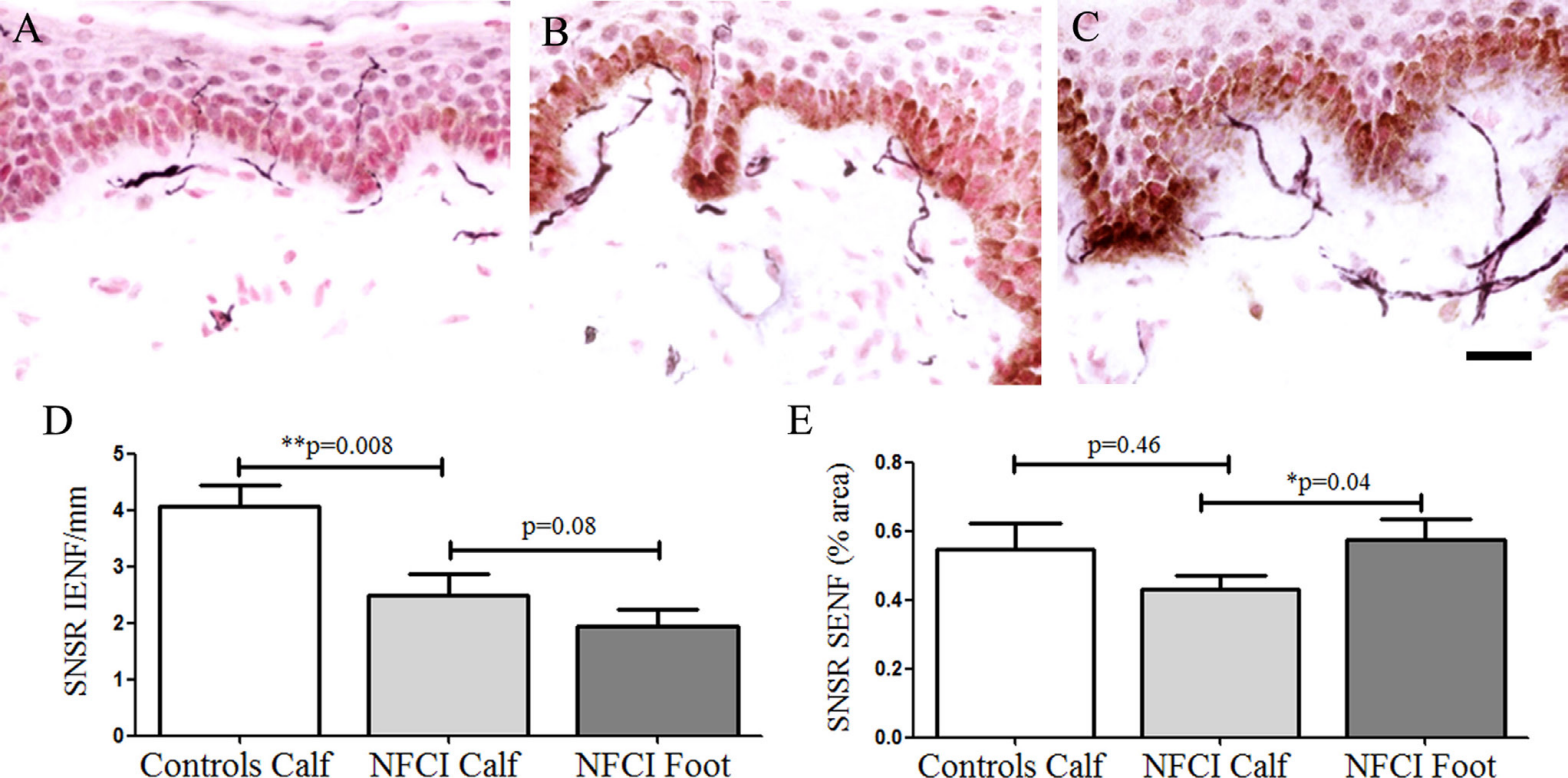
Sensory neuron-specific receptor (SNSR) immunoreactivity in skin. SNSR staining in control calf skin **(A)**, in non-freezing cold injury (NFCI) calf skin **(B)**, and in NFCI foot skin **(C)**; scale bar = 50 µm; bar charts of the SNSR intraepidermal nerve fibre (IENF) fibres/mm **(D)**; bar charts of the image analysis of the SNSR subepidermal nerve fibre (SENF) (% area) **(E)**.

Sensory neuron-specific receptor IENF was decreased in NFCI skin (*p* = 0.008; Figure 5). IENF and SENF analysis showed 90 and 80%, respectively, of NFCI subjects had abnormal values.

Sensory neuron-specific receptor IENF fibres in NFCI foot skin were not significantly different to NFCI calf skin (*p* = 0.08, Figure 5). There was a significant increase of SNSR SENF in NFCI foot skin compared to the NFCI calf skin (*p* = 0.03; Figure 5).

#### Growth-Associated Protein 43

**Figure 6:**
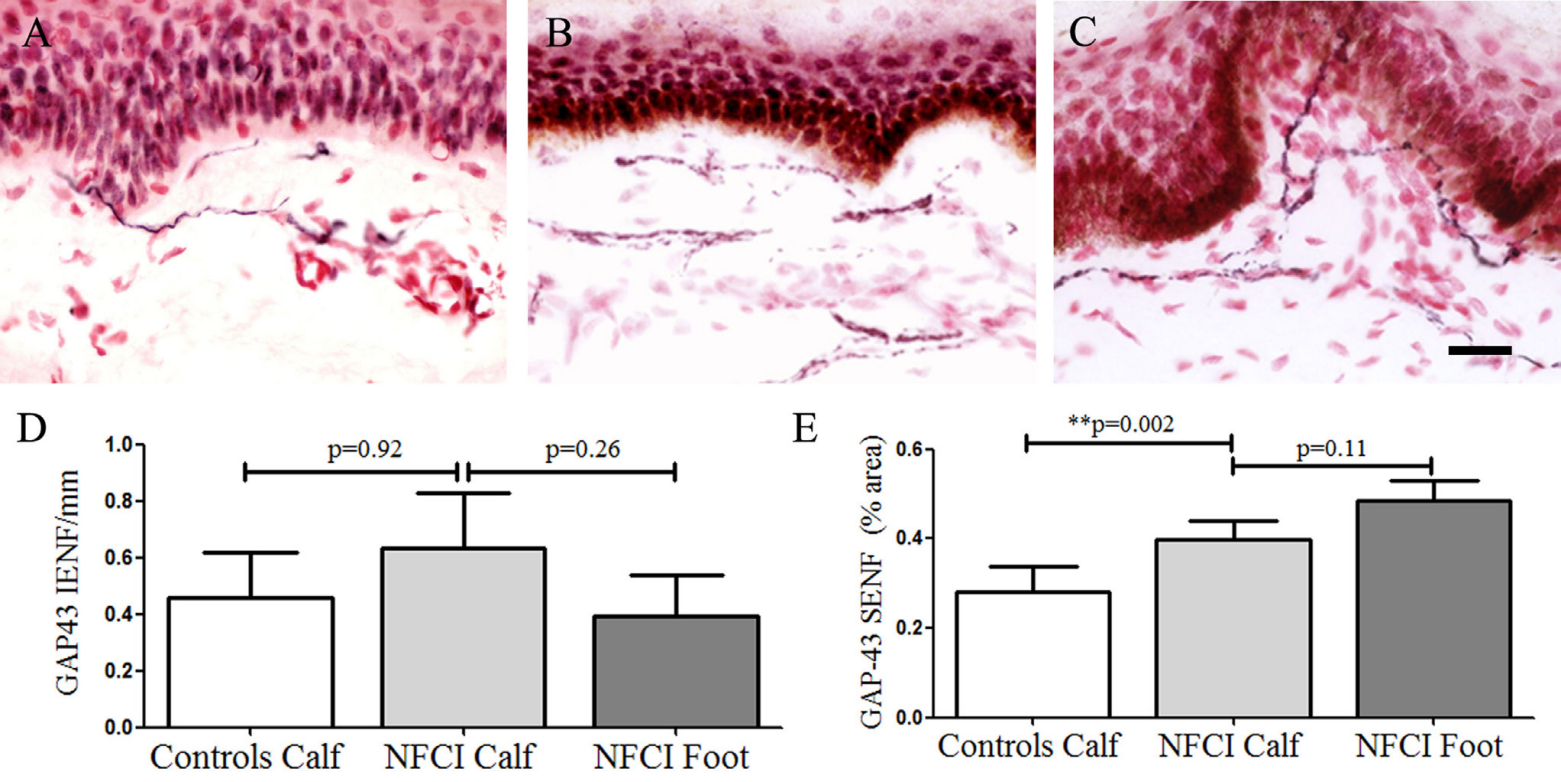
Growth-associated protein 43 (GAP43) immunoreactivity in skin. GAP43 staining in control calf skin **(A)**, in non-freezing cold injury (NFCI) calf skin **(B)**, and in NFCI foot skin **(C)**; scale bar = 50 µm; bar charts of the GAP43 intraepidermal nerve fibre (IENF) fibres/mm **(D)**; bar charts of GAP43 subepidermal nerve fibre (SENF) (% area) **(E)**.

Growth-associated protein 43 IENF in calf skin showed a trend for an increase in NFCI (*p* = 0.92; Figure 6). GAP43 SENF in NFCI calf skin showed a significant increase (*p* = 0.002; Figure 6). IENF and SENF analysis showed 83.3 and 76.6%, respectively, of NFCI subjects, had abnormal values in calf skin.

GAP43 IENF in NFCI foot skin was not significantly different to NFCI calf skin (*p* = 0.26; Figure 6). GAP43 SENF in NFCI foot was also not different to NFCI calf skin (*p* = 0.11; Figure 6).

#### von Willebrand Factor

**Figure 7:**
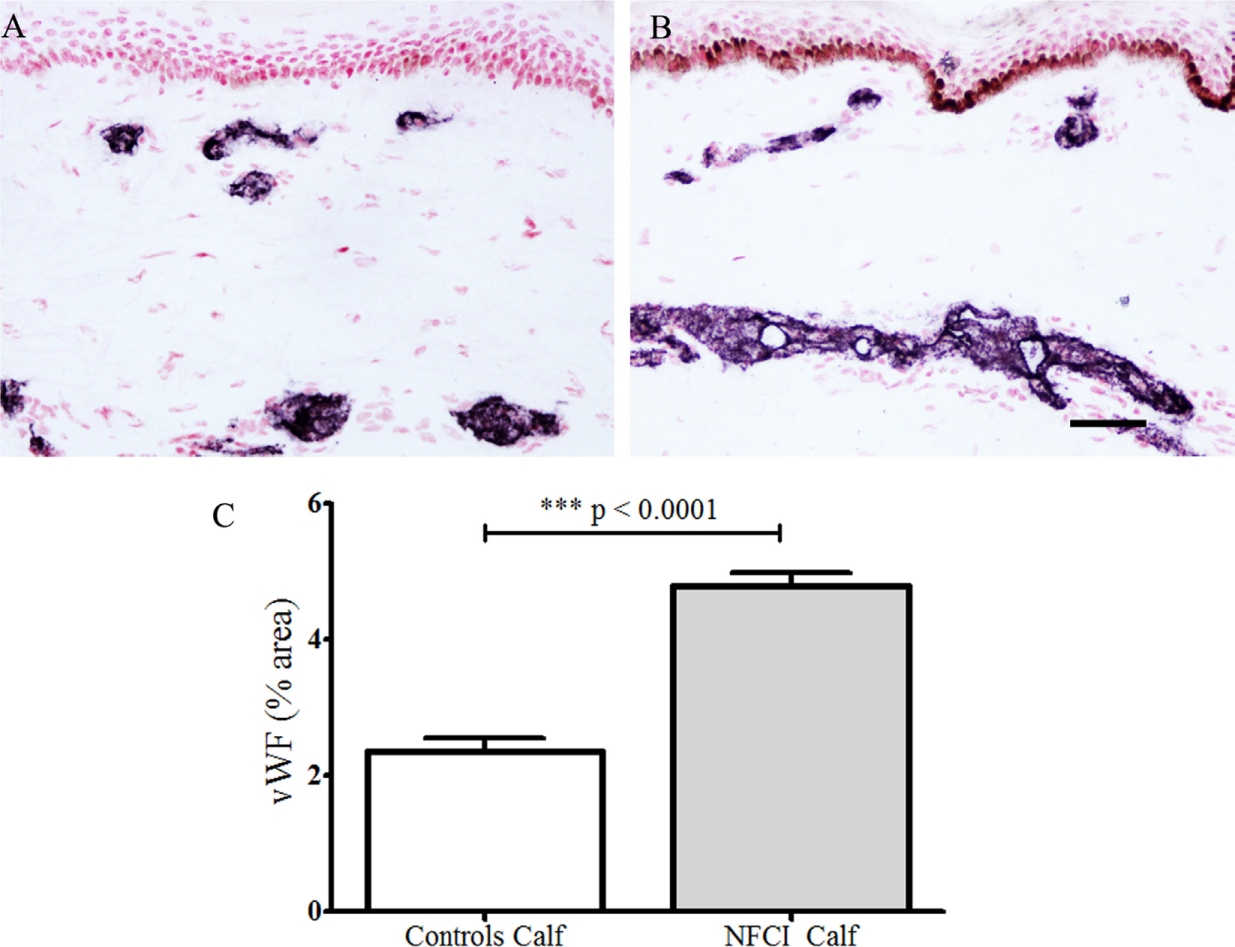
von Willebrand Factor (vWF) immunoreactivity in skin. vWF staining in control calf skin **(A)** and in non-freezing cold injury (NFCI) calf skin **(B)**; scale bar = 100 µm; bar chart of the image analysis of the vWF immunoreactivity **(C)**.

Antibodies to vWF stained blood vessels (Figure 7). There was a statistically significant increase of % area in NFCI calf skin (*p* < 0.0001; Figure 7). All soldiers (100%) had abnormal values for vWF in calf skin.

#### Vascular Endothelial Growth Factor

**Figure 8:**
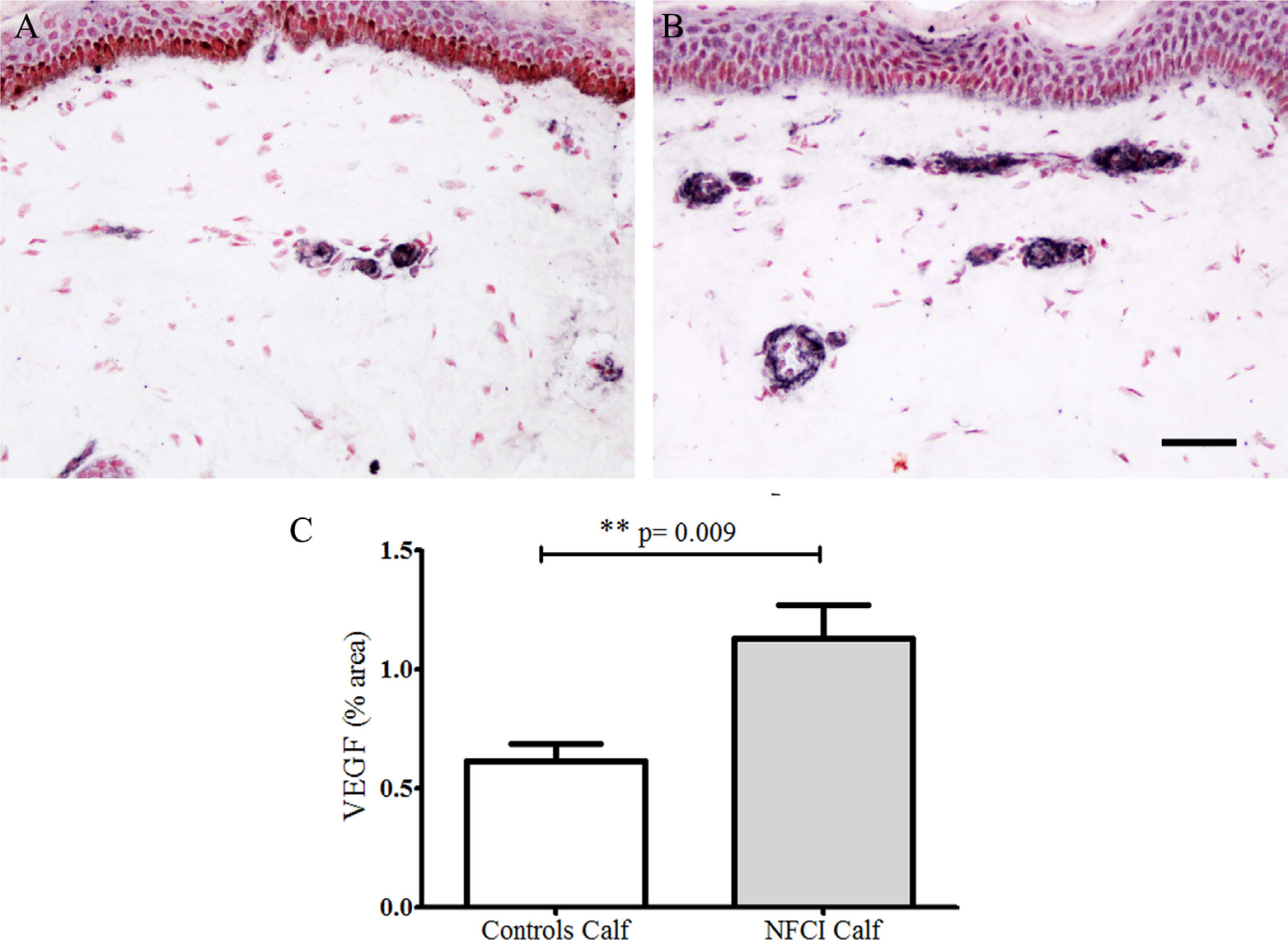
Vascular endothelial growth factor (VEGF) immunoreactivity in skin. VEGF staining in control calf skin **(A)** and in non-freezing cold injury (NFCI) calf skin **(B)**; scale bar = 100 µm; bar chart of the image analysis of the VEGF immunoreactivity **(C)**.

Antibodies to VEGF in calf skin stained blood vessels in control and NFCI skin (Figure 8). There was a statistically significant increase in the % area of VEGF in NFCI calf skin (*p* = 0.009; Figure 8). 94% of NFCI subjects had abnormal values for VEGF in calf skin.

#### Endothelial Nitric Oxide Synthase

**Figure 9:**
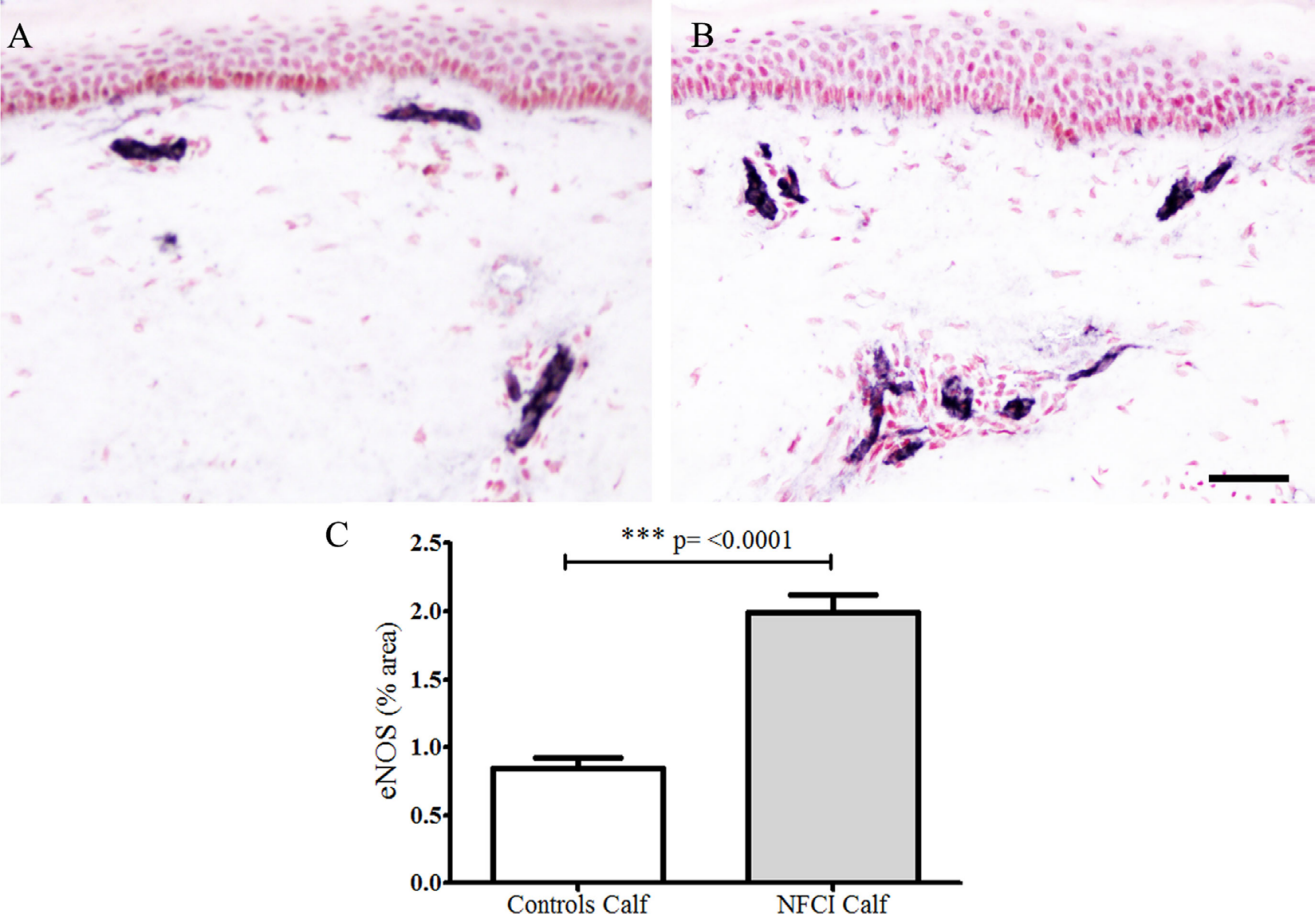
Endothelial nitric oxide synthase (eNOS) immunoreactivity in skin. eNOS staining in control calf skin **(A)** and in non-freezing cold injury (NFCI) calf skin **(B)**; scale bar = 100 µm; bar chart of the image analysis of the eNOS immunoreactivity **(C)**.

Antibodies to eNOS in calf skin stained blood vessels in control and NFCI skin (*n* = 19; Figure 9). There was a statistically significant increase in % area of eNOS in NFCI calf skin (*p* < 0.0001; Figure 9). All soldiers (100%) had abnormal values for eNOS in calf skin.

#### Calcitonin Gene-Related Peptide

**Figure 10:**
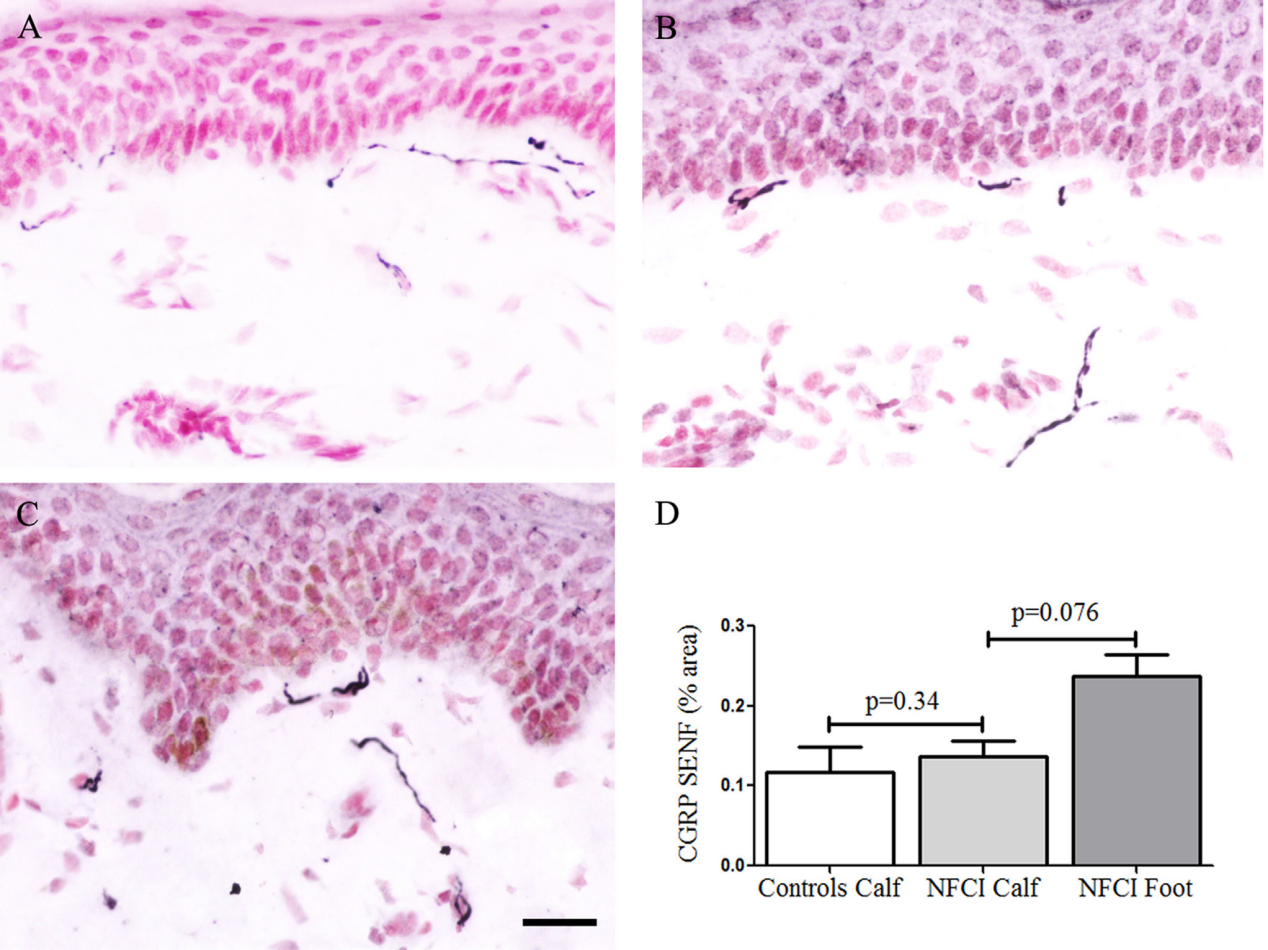
Calcitonin gene-related peptide (CGRP) immunoreactivity in skin. CGRP staining in control calf skin **(A)**, in non-freezing cold injury (NFCI) calf skin **(B)**, and in NFCI foot skin **(C)**; scale bar = 50 µm. Bar chart of the image analysis of the CGRP subepidermal nerve fibre (SENF) (% area) **(D)**.

There were few IENF observed for the neuropeptide CGRP, and these were not counted. Image analysis of CGRP SENF in NFCI calf skin (*n* = 16) and NFCI foot skin (*n* = 10) showed no difference in NFCI calf skin (Figure 10), there was a trend for an increase in CGRP SENF in NFCI foot compared to NFCI calf skin (*p* = 0.076; Figure 10).

#### Transient Receptor Potential Cation Channel, Subfamily A Member 1

**Figure 11:**
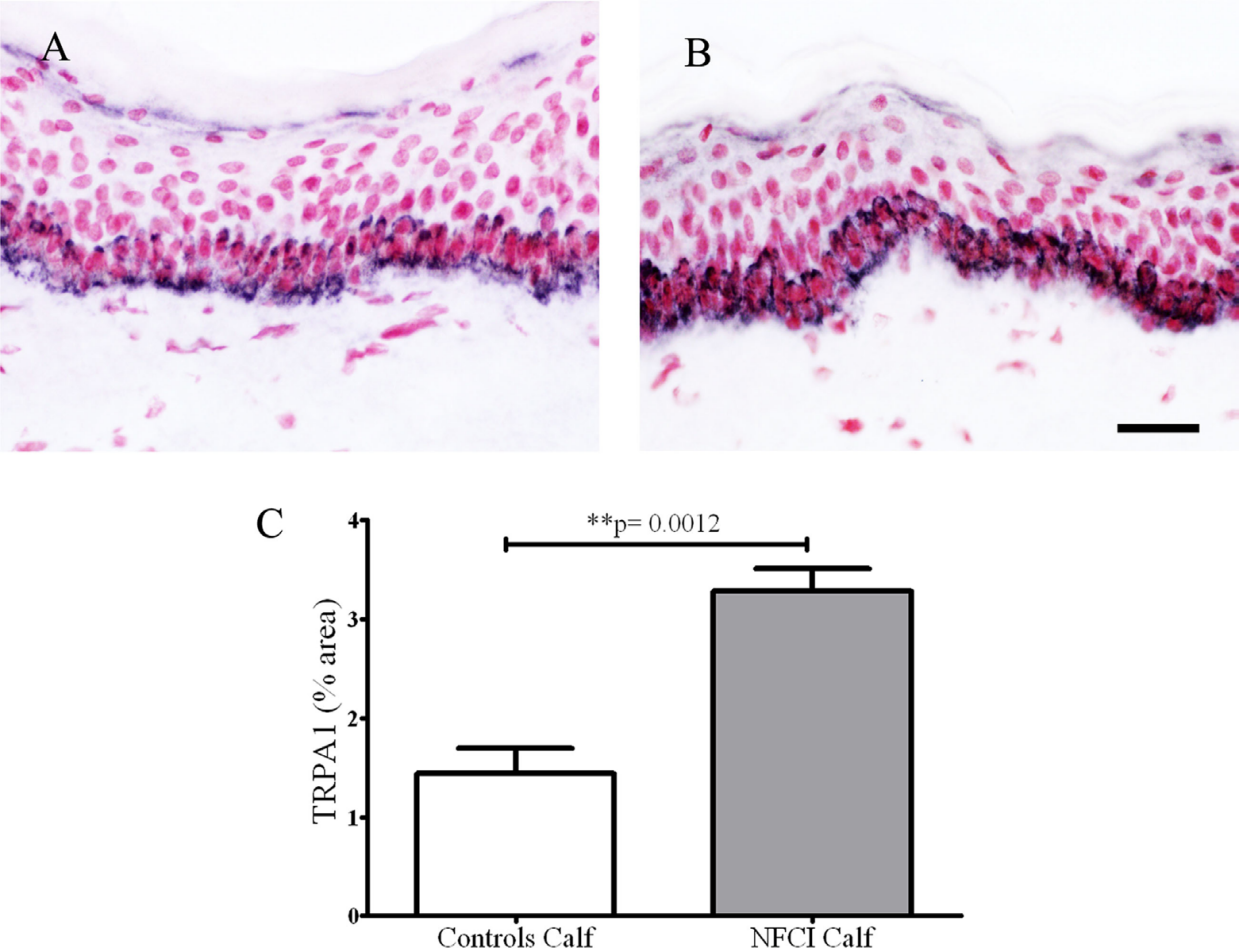
Transient receptor potential cation channel, subfamily A member 1 (TRPA1) immunoreactivity in skin. Basal keratinocyte cell staining with TRPA1 in control calf skin **(A)** or non-freezing cold injury (NFCI) calf skin **(B)**; scale bar = 50 µm. Bar chart of the image analysis of the TRPA1 immunoreactivity **(C)**.

Transient receptor potential cation channel, subfamily A member 1 antibody labelled basal keratinocytes of the epithelium in control and NFCI calf skin (*n* = 7) (Figures 11A,B). Image analysis showed a statistically significant increase in % area in NFCI calf skin (*p* = 0.0012; Figure 11C). TRPA1-positive nerve fibres were too sparse to quantify.

#### P2X7 Receptor

**Figure 12:**
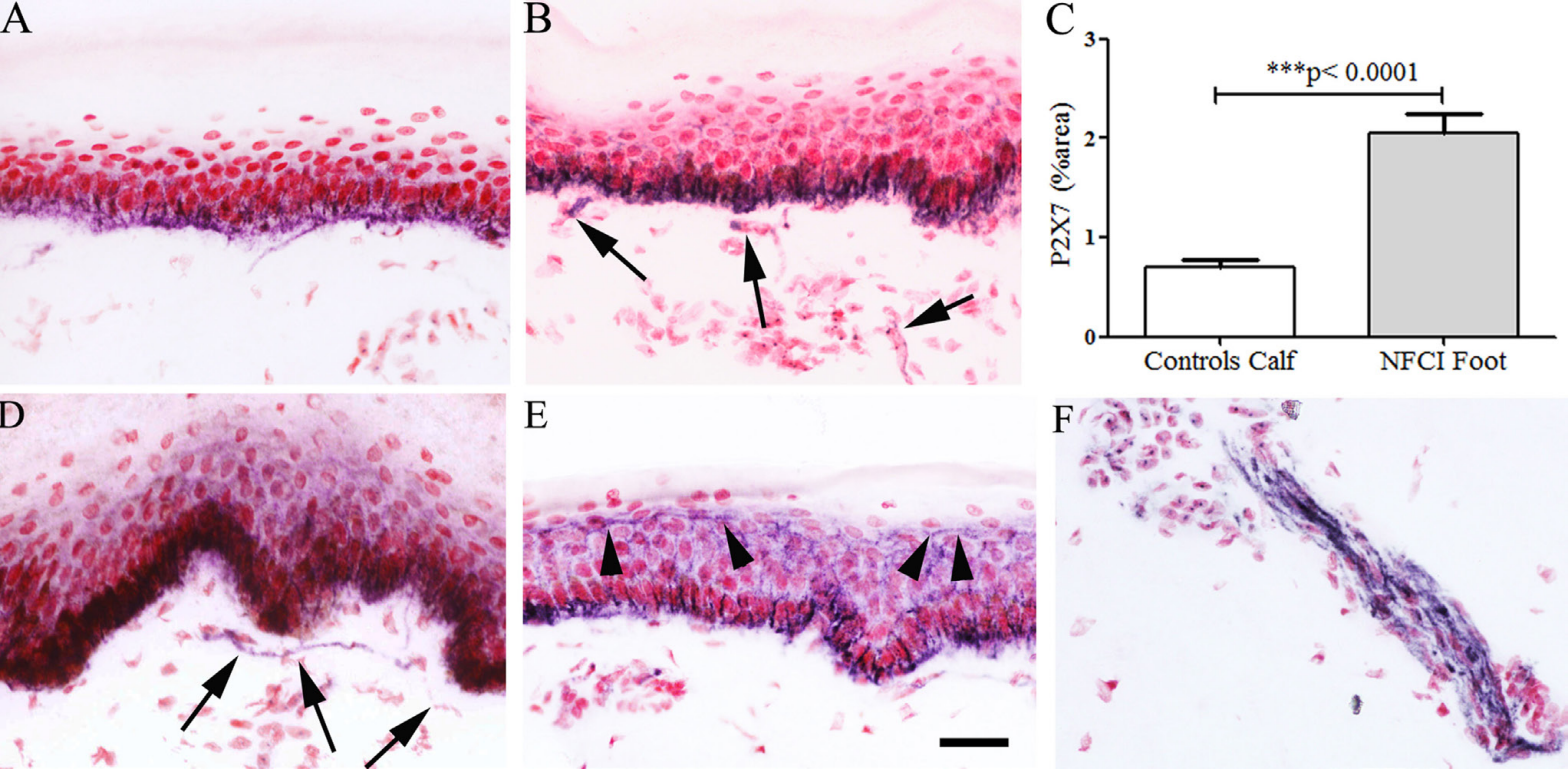
P2X purinoceptor 7 (P2X7) receptor immunoreactivity in skin. Keratinocyte and nerve fibre (arrowed) staining in control calf skin **(A)** and non-freezing cold injury (NFCI) calf skin **(B)**; bar chart of the image analysis of P2X7 receptor epidermal immunoreactivity in calf skin **(C)**; P2X7 receptor immunoreactive nerve fibres (arrowed) in the subepidermal region **(D)**, and suprabasal keratinocyte staining (arrowheads) to the top of the epidermis, in NFCI foot skin **(E)**; P2X7 receptor nerve fibre bundle in deep dermis of foot skin **(F)**; scale bar = 50 µm.

Image analysis of keratinocyte P2X7 receptor immunoreactivity showed a significant increase in NFCI calf skin (*n* = 10, *p* < 0.0001; Figure 12). The presence of suprabasal staining, up to the top of the epidermis, was observed in NFCI skin, but not control skin. P2X7-positive nerve fibres were observed in subepidermal and deep dermis regions of NFCI skin, but were too sparse to quantify.

### Colocalisation of PGP 9.5 or SNSR Neuronal Staining with the Endothelial Cell Marker CD31

**Figure 13:**
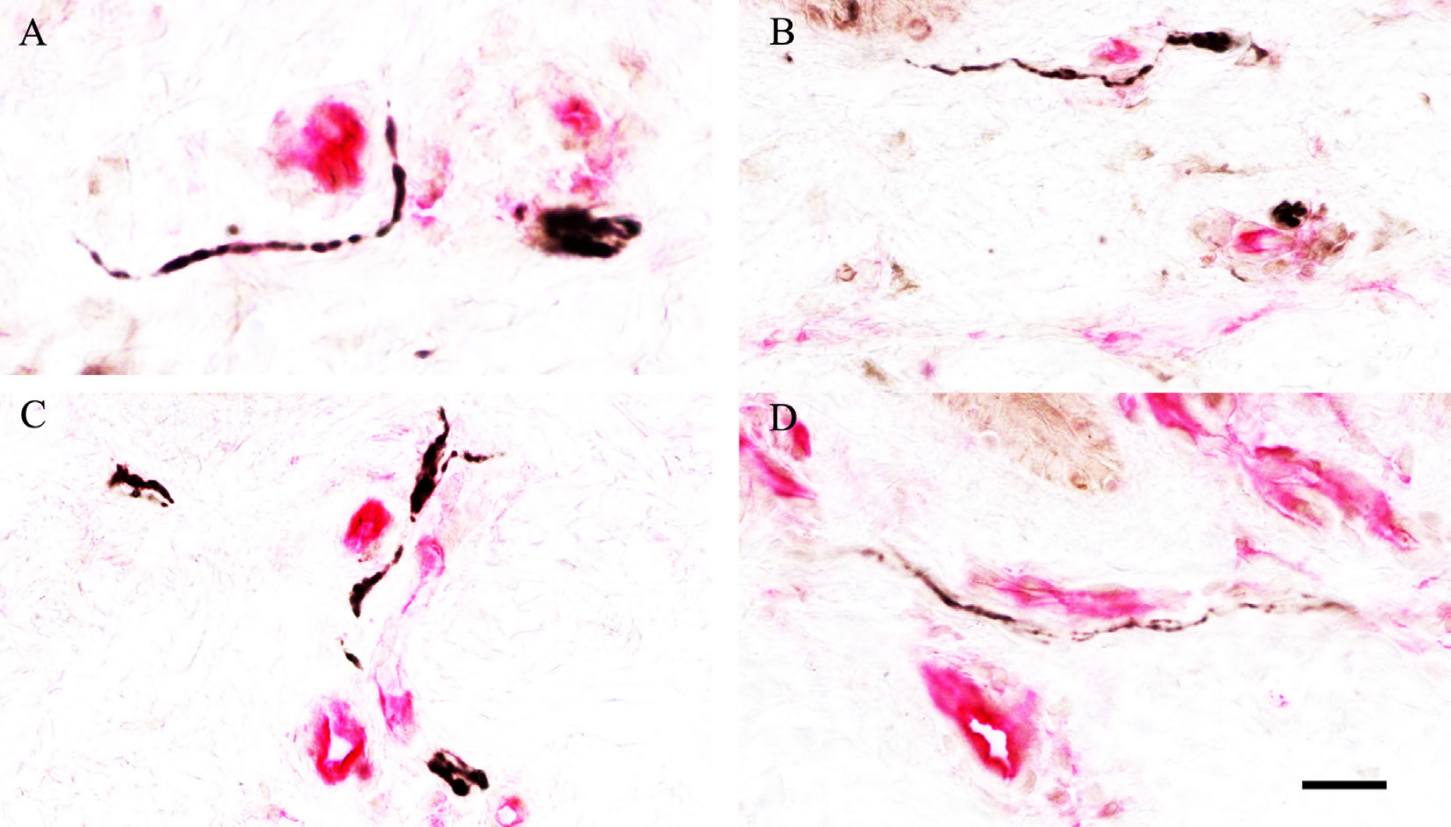
Neuronal and vascular colocalisation staining in skin. Association of protein gene product 9.5 **(A,B)** or sensory neuron-specific receptor **(C,D)** neuronal staining (black), with the blood vessel endothelial cell marker cluster of differentiation 31 (red), in the subepidermis of non-freezing cold injury foot skin; scale bar = 50 µm.

Colocalisation PGP 9.5 or SNSR neuronal staining with the vascular endothelial cell marker CD31 showed a strong association between nerve fibres and the vasculature in the subepidermal region (Figure 13).

### Ratio of SENFs and Vascular Markers

**Figure 14:**
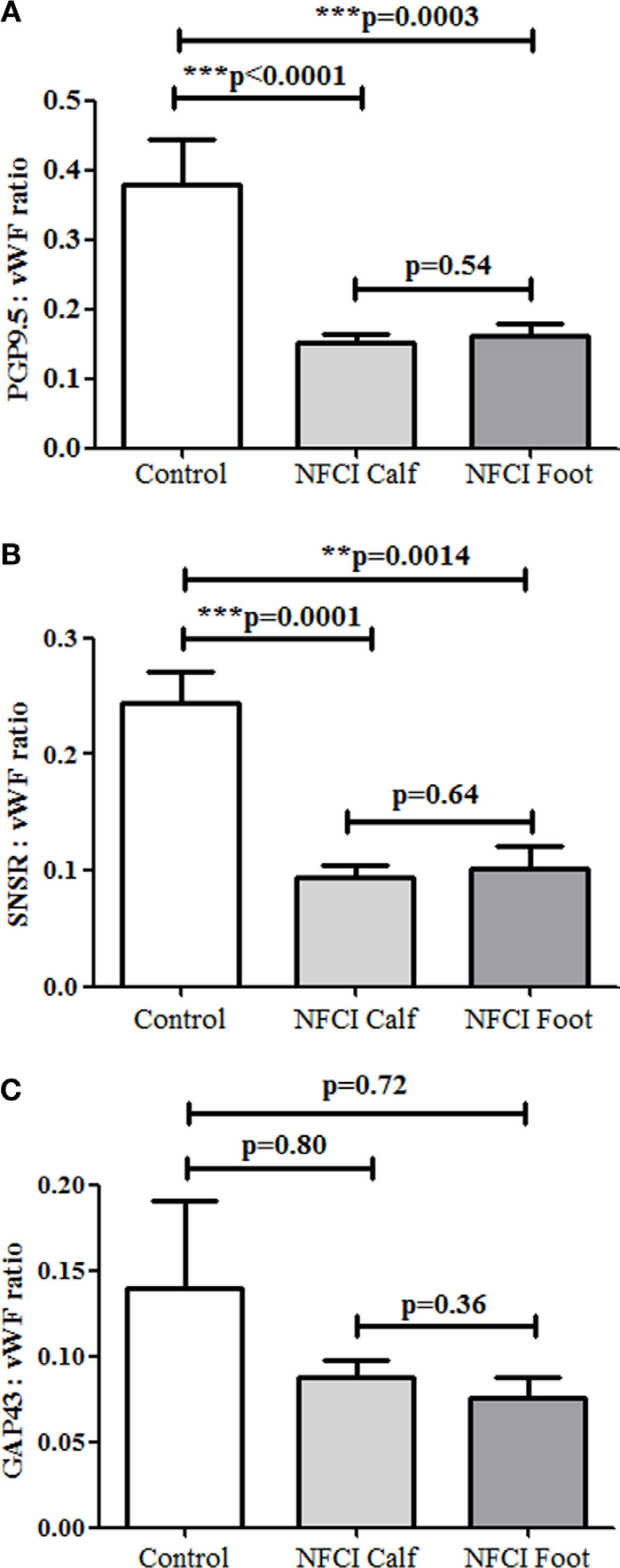
Ratios of subepidermal nerve fibres (SENFs) to von Willebrand factor (vWF) in non-freezing cold injury (NFCI) skin. Ratios of nerve markers to vWF, **(A)** protein gene product 9.5 (PGP 9.5) (PGP 9.5:vWF) in control and NFCI calf skin, **(B)** sensory neuron-specific receptor (SNSR) (SNSR:vWF), and **(C)** growth-associated protein 43 (GAP43) (GAP43:vWF).

Statistical analysis of the ratio between nerve markers and vWF showed the ratios for PGP:vWF and SNSR:vWF were statistically lower in both calf and foot skin (Figure 14).

## Discussion

The patients we assessed had documented exposure to cold and wet conditions, and sought medical attention soon thereafter, thereby reporting sensory symptoms in affected limbs. Persistent cold hypersensitivity was reported by most patients. Clinical examination was unremarkable, other than loss of joint position sense at the great toe in a few subjects, and of pinprick sensation, which was abnormal in the feet of 67% patients. However, these are not objective assessments, or as useful as the other measures described below.

### Neurophysiological Assessments: QST and Nerve Conduction

Sensory nerve fibre function assessment with QST in the feet showed abnormal monofilament thresholds (63%), vibration thresholds (40%), cool thresholds (73%), warm thresholds (83%), and cold and heat pain thresholds (66%). However, QST is subjective and may be difficult to perform for some patients with severe limb pain and cutaneous hypersensitivity. Nerve conduction studies are objective and showed axonal neuropathy in 23% subjects, affecting only the plantar nerves in the feet. Our findings indicate that plantar nerve conduction studies are a useful and necessary part of assessment in NFCI, to document involvement of large sensory nerve fibres. SSRs recorded from both palms and soles were found to be normal.

Previous publications have described these tests in patients who had a freezing cold injury. Decreased nerve conduction velocity (31) and features of axonal neuropathy have been reported, and normal SSR (32, 33). Cold-evoked symptoms and abnormalities of sensory thresholds were detected with QST in patients with cold exposure injury (34–36).

### Skin Biopsy Assessments

In our cohort, 27 patients (90%) had abnormal IENF values, and 28 (93%) abnormal SENF values, in calf skin for PGP 9.5; the remaining patients had abnormalities of other nerve markers, and 1 had abnormal nerve conduction study. Skin biopsy assessment with a range of markers is therefore the assessment of choice in such patients. Sensory nerve sub-set markers TRPV1 and SNSR IENF were found to be decreased. PGP 9.5, SNSR, CGRP, and GAP43 were reduced in the subepidermal region of calf skin, but relatively preserved in foot skin (whereas they are normally fewer in the more distal limb regions). SENF GAP43 was increased in calf skin, and similarly in foot skin, suggesting that nerve regeneration was prominent.

Two previous case reports involving severe cold injury (32, 37) have reported PGP 9.5 nerve fibre loss in skin, though in one case there was marked tissue damage, and in the other profound global hypothermia with generalised nerve dysfunction over months.

In our cohort, skin biopsies showed a marked increase of blood vessels, as indicated by the immunostaining for vWF, and also VEGF and eNOS. The increased VEGF may reflect the changes secondary to hypoxia and ischaemia with formation of new blood vessels (38), which in NFCI may be induced by cold and mechanical pressure. The increased expression of nitric oxide synthase may be related to functional changes in increased blood vessels (39).

In our cohort, colocalisation of PGP 9.5, SNSR, and endothelial marker for CD31 showed association between nerve fibres and the vasculature in the subepidermal region. The ratio of nerve markers to vWF was decreased for PGP 9.5 and SNSR in NFCI calf skin. The increased vascularity, and its ratio to adjacent nerve fibres, may play an important role in patients who show neuropathic symptoms, such hypersensitivity and pain. We recently showed in patients with advanced diabetic polyneuropathy that increased blood vessels and decreased ratio to SENF distinguished painful from non-painful neuropathy, while IENF and SENF were similarly reduced overall in both groups, as expected (18). Relative excess of target-derived nerve growth factor (NGF) (from blood vessels and/or keratinocytes) may lead to neuropathic pain (40). Thus, at this stage of NFCI, decreased regional density of nerve fibres and increased blood vessels and associated regenerating nerve fibres may all contribute to the clinical symptomatology and phenotype (pain, cold hypersensitivity, and elevated sensory thresholds).

Basal keratinocytes secrete neurotrophic factors NGF and GDNF, which regulate IENF nociceptor structure and function in health and disease (40), and express the cold sensor TRPA1 (19, 41). We found an increased level of TRPA1 basal cell staining in NFCI calf skin, and also in suprabasal keratinocytes. While speculative, the TRPA1 overexpression in keratinocytes may in part underlie persistent cold hypersensitivity *via* release of NGF, and its blockade may prevent or treat this symptom. Increased purinoreceptor P2X7 expression was also found in basal keratinocytes and may contribute to the pathophysiology, as it is involved in key pain mechanisms (20, 42, 43).

### Pathophysiological Mechanisms

Our study suggests that changes in the innervation pattern of the skin, increased vascularity, and keratinocyte expression of ion channels may all play a role in the pathogenesis of NFCI, thereby leading to a chronic painful vaso-neuropathy. The sequence of events in the stages of NFCI in relation to these putative mechanisms, and the long-term outcomes, deserve further study.

While freezing cold injury results from the cryogenic insult directly to cells along with vascular stasis and anoxia (44), NFCI originates from an impairment of the tissue function resulting in minor degrees of initial tissue damage and long-lasting sequelae such as cold hypersensitivity (45). Friedman attributed disturbance of the microcirculation as the initial event, leading to stagnation of blood, followed by thrombosis and gangrene (12, 13).

Following cold exposure, autoregulatory mechanisms lead to an initial vasoconstriction, aimed to maintain the body temperature (46), along with concomitant metabolic changes (47, 48). During persisting cold exposure, the initial vasoconstriction is followed by a compensatory vasodilatation, which reduces the initial decrease in blood flow (49, 50), i.e., cold-induced vasodilatation (CIVD) preserves the vitality of tissues. Subjects prone to NFCI may show an abnormal CIVD (51, 52). Changes in the microcirculatory autoregulation mechanisms have also been considered responsible for the increased cold sensitivity (53, 54). NFCI has been reported more frequently in soldiers with Afro-Caribbean ethnicity (55, 56), as in our cohort, and these underlying mechanisms deserve further study.

If the cold exposure is intense and prolonged, both ischaemia and reperfusion (57, 58) can cyclically alternate. The multiple cycles of ischaemia–reperfusion have been shown to facilitate the cold nerve injury *via* reactive oxygen species (59), accompanied by the disruption of the integrity of the small blood vessels, that progressively aggravates the initial nerve damage (60). The changes observed after a repeated NFCI have been shown to resemble those observed during ischaemia/reperfusion injury (61). Animal models confirmed the involvement of both myelinated and unmyelinated fibres (11, 58, 62, 63), and supported the ischaemic origin of the nerve injury (64).

## Conclusion

To our knowledge, this is the first study providing clear evidence of the involvement of cutaneous small nerve fibres and blood vessels in a cohort of patients with mild NFCI. Our findings suggest that increased blood vessels following tissue ischaemia/hypoxia, with disproportionate density of associated nerve fibres, may underlie pathogenesis of NFCI, leading to a “painful vaso-neuropathy.” Further studies in a larger cohort of patients and control subjects, including pathophysiological, genetic, and epigenetic mechanisms, are indicated.

## Ethics Statement

In this manuscript, we report the assessment of 30 soldiers who were referred by UK medical practitioners to our hospital NHS (National Health Service) clinic, with a provisional diagnosis of NFCI. They were assessed at the Peripheral Neuropathy Clinic, Hammersmith Hospital, Imperial College Healthcare NHS Trust, according our routine NHS Trust clinical practice pathway and investigations. All soldiers gave their written informed consent for skin biopsy. The data presented here are the outcome of an internal Departmental audit, which reviewed the assessment of such patients, and the usefulness of our tests. This has been stated in the manuscript (Materials and Methods, Participants, line 92–100).

## Author Contributions

PA and RB organized patient referrals and coordination of assessments; RP helped with clinical assessments; PM performed the nerve conduction studies; YY and PD analyzed the skin biopsies. All authors contributed to drafting the manuscript and approved the final manuscript.

## Conflict of Interest Statement

The authors declare that the work was conducted in the absence of any commercial or financial relationships that could be construed as a potential conflict of interest. The reviewer DM and handling editor declared their shared affiliation.

## Abbreviations

NFCI, non-freezing cold injury; CIVD, cold-induced vasodilatation; NCS, nerve conduction study; DPN, diabetic polyneuropathy; PGP 9.5, protein gene product 9.5; TRPV1, transient receptor potential cation channel subfamily V member 1; TRPA1, transient receptor potential cation channel, subfamily A member 1; SNSR, sensory neuron-specific receptor; GAP43, growth-associated protein 43; vWF, von Willebrand factor; VEGF, vascular endothelial growth factor; eNOS, endothelial nitric oxide synthase; CGRP, calcitonin gene-related peptide; P2X7, P2X purinoceptor 7; CD31, cluster of differentiation 31.
